# Functional roles and redundancy of demersal Barents Sea fish: Ecological implications of environmental change

**DOI:** 10.1371/journal.pone.0207451

**Published:** 2018-11-21

**Authors:** Magnus Aune, Michaela M. Aschan, Michael Greenacre, Andrey V. Dolgov, Maria Fossheim, Raul Primicerio

**Affiliations:** 1 Akvaplan-niva AS, The Fram Centre, Tromsø, Norway; 2 Norwegian College of Fishery Science, UiT–The Arctic University of Norway, Tromsø, Norway; 3 Department of Economics and Business, Universitat Pompeu Fabra and Barcelona Graduate School of Economics, Ramon Trias Fargas, Barcelona, Spain; 4 Knipovich Polar Research Institute of Marine Fisheries and Oceanography, Murmansk, Russian Federation; 5 Institute of Marine Research, The Fram Centre, Tromsø, Norway; Aristotle University of Thessaloniki, GREECE

## Abstract

When facing environmental change and intensified anthropogenic impact on marine ecosystems, extensive knowledge of how these systems are functioning is required in order to manage them properly. However, in high-latitude ecosystems, where climate change is expected to have substantial ecological impact, the ecosystem functions of biological species have received little attention, partly due to the limited biological knowledge of Arctic species. Functional traits address the ecosystem functions of member species, allowing the functionality of communities to be characterised and the degree of functional redundancy to be assessed. Ecosystems with higher functional redundancy are expected to be less affected by species loss, and therefore less sensitive to disturbance. Here we highlight and compare typical functional characteristics of Arctic and boreal fish in the Barents Sea and address the consequences of a community-wide reorganization driven by climate warming on functional redundancy and characterization. Based on trait and fish community composition data, we assessed functional redundancy of the Barents Sea fish community for the period 2004–2012, a period during which this northern region was characterized by rapidly warming water masses and declining sea ice coverage. We identified six functional groups, with distinct spatial distributions, that collectively provide a functional characterization of Barents Sea fish. The functional groups displayed different prevalence in boreal and Arctic water masses. Some functional groups displayed a spatial expansion towards the northeast during the study period, whereas other groups showed a general decline in functional redundancy. Presently, the observed patterns of functional redundancy would seem to provide sufficient scope for buffering against local loss in functional diversity only for the more speciose functional groups. Furthermore, the observed functional reconfiguration may affect future ecosystem functioning in the area. In a period of rapid environmental change, monitoring programs integrating functional traits will help inform management on ecosystem functioning and vulnerability.

## Introduction

Marine ecosystems presently undergo substantial compositional and structural alterations in response to environmental change, with associated implications for ecosystem functioning and vulnerability [1]. Although climate is predicted to change most rapidly at high-latitudes [2], where climate-induced ecological responses are already evident [3, 4], Arctic marine ecosystems are generally understudied [5], and very little research has been directed towards ecosystem functioning and possible alterations.

Marine ecosystem functioning is influenced by species composition via the functional traits (i.e., properties) of individual species. Functional traits allow the identification of the ecosystem functions of individual community members, the functional characterization of a community and the assessment of its functional diversity and redundancy [6, 7, 8]. In ecosystems, functional diversity (FD) and functional redundancy (FR) are important components of community vulnerability [9]. FD can be regarded as a measure of the number of properties that an assemblage of species possesses, therefore representing the number of ecosystem properties that the assemblage provides to the ecosystem. FR informs about the number of species that play similar functional roles in a community [8]. In ecosystems, a mix of various functional types of species (i.e., high FD) is necessary in order to ensure sufficient adaptability when facing environmental perturbations [9] as a higher number of functional “tools” increases the capacity to cope with upcoming environmental challenges. Assessing the functional character and the diversity and redundancy of a community will therefore allow ecosystem functioning and vulnerability to be addressed. As such, FR informs about the vulnerability of the ecosystem. High FR implies that species loss might not necessarily induce any clear, immediate effects on ecosystem functioning, since similar species could compensate functionally [10]. Thus, FR is thought to influence buffering capacity, and therefore the ability of ecosystems to maintain their functionality when facing stress [10, 11].

In this paper, we provide a functional classification of the fish community in the Barents Sea, a sub-Arctic shelf sea off the northern coasts of Russia and Norway. The Barents Sea encompasses both Arctic and Atlantic water masses, inhabited by different fish species [12] characterized by different traits [13, 14]. The area is ideal to investigate whole-community structure and community-wide shifts; it is well-studied, with an extensive database, and has strong, natural gradients in environmental characteristics. Furthermore, recent climate warming has resulted in an increase of warm Atlantic water and a decrease of cold Arctic water, and a decline in sea ice coverage in the area ([15, 16], and citations therein). In recent years, these environmental perturbations have caused structural reorganisation in the fish community and its trophic configuration (i.e., *borealization*) [12, 17] and associated spatial alterations in FD [13].

Here, we analyse the spatial patterns in the redundancy of functional groups, and investigate the recent trends in FR associated with documented poleward distributional shifts driven by rapid climate warming. Based on functional traits data, we allocate fish species to functional groups. This information is then integrated with fish composition data, sampled annually from the entire Barents Sea during the period 2004–2012, to assess the spatial distribution and redundancy of each functional group. We use the following two hypotheses:

- The fish communities in Arctic and Atlantic waters of the Barents Sea are dominated by different functional groups, and therefore different functional processes.
- Recent changes in fish community structure driven by warming have implications for the FR in time and space.

## Materials and methods

### Study area

**Fig 1:**
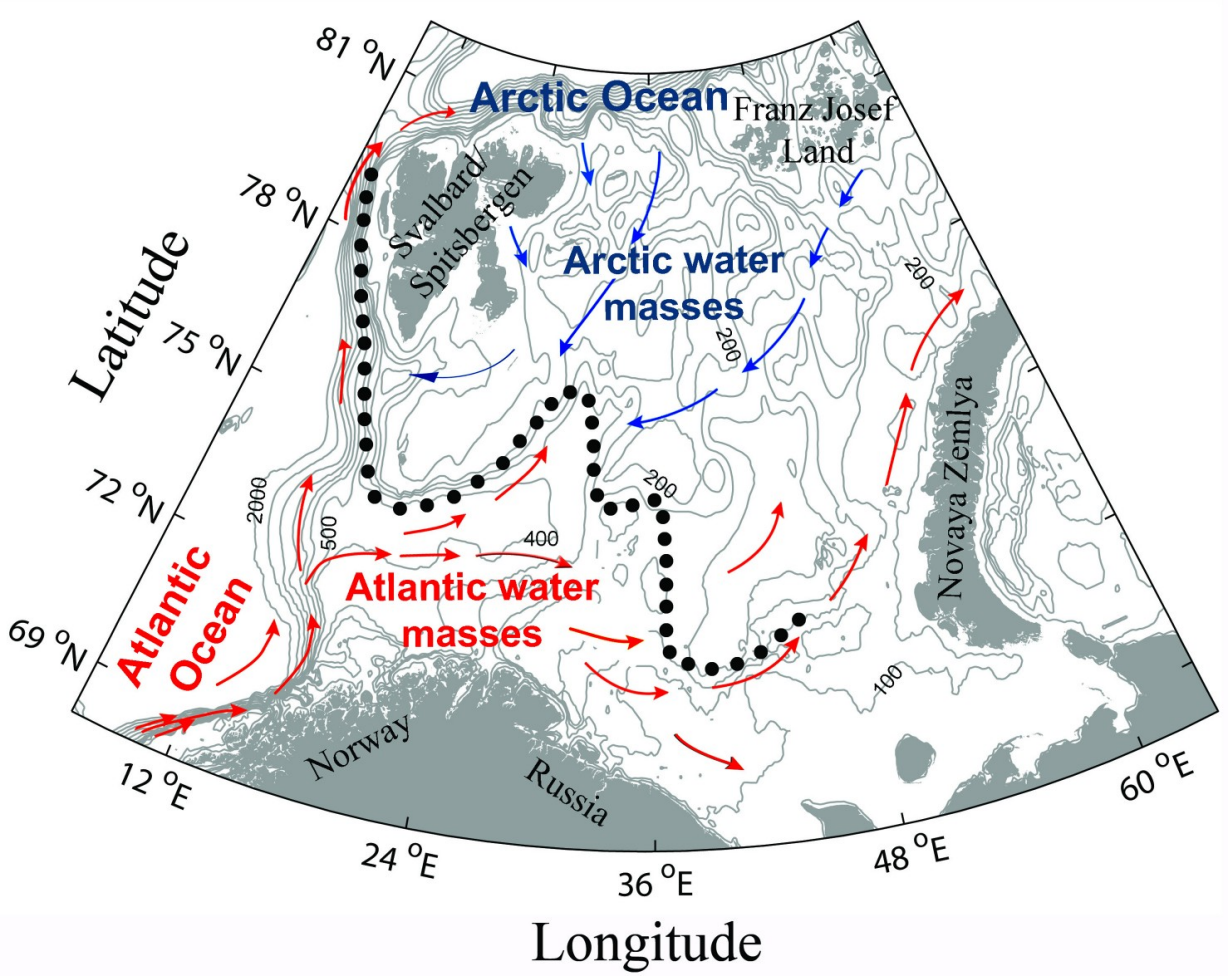
Map of the Barents Sea, based on Loeng [19]. Red arrows indicate Atlantic water currents, blue arrows indicate Arctic water currents, and dotted line indicates approximate position of the Polar Front.

The Barents Sea is a shallow shelf sea (average depth approximately 230 m), delimited by the shelf break to the Atlantic Ocean and Svalbard/Spitzbergen archipelago in the west, the shelf break to the Arctic Ocean in the north, the Novaya Zemlya archipelago to the east and the coasts of Norway and Russia to the south (Fig 1). The area comprises both Atlantic and Arctic water masses, which mix at the Polar Front (PF) (see dotted line in Fig 1 for approximate position). From the south-west, warm Atlantic water masses flow into the Barents Sea, and the bottom temperatures here vary between 3.5–7.5°C, depending on the season [18]. In Atlantic water masses the salinity is typically 35.0 [18]. This inflow of Atlantic water determines, to a large degree, the climate of the region [19]. A southward flow of cold Arctic water provide an Arctic environment in the north and north-east, with temperatures generally below 0°C and salinity in the range 32.0–34.8 [18]. In the western part of the Barents Sea, the PF is topographically steered and relatively stable; in the eastern Barents Sea, the constraints due to the bottom topography are weaker and the PF is broader and less well defined [19]. The position of the PF displays a strong inter-annual and a weaker seasonal variability [20]. Furthermore, recent evidence suggests that the PF divides into a northern and a southern branch in the eastern Barents Sea [20]. Although the PF is a prominent feature of the water masses in the Barents Sea, it therefore remains challenging to conduct explicit analyses of spatial ecological patterns in relation to the position of the PF. The sea ice cover is at a maximum in early spring, generally covering the areas north of the PF, retreating northwards and eastwards from April until September [18]. In recent years warming of the Barents Sea water masses has occurred, mainly due to increased Atlantic water heat transport [16, 21]. This has resulted in an increase in the proportion of warm Atlantic water and a decline in the proportion of cold Arctic water in the Barents Sea [15, 16]. This heating is the main reason for the declining sea ice coverage in the northern parts of the Barents Sea [16], a pattern that is common for the Arctic Ocean in general [22]. A significant spatial variation in primary production has also been documented in the Barents Sea, average annual primary production being the highest (160 g C m^-2^ yr^-1^) in the Atlantic water masses in the south-west, decreasing towards the north and northeast (to 60 g C m^-2^ yr^-1^). Consequently, the flux of organic matter is also highest in the south-west and lower in the north and northeast. However, in the Arctic water masses, the proportion of primary production that settles to the bottom is much higher than in the south-west (53% vs. 27%) [23], which is plausibly reflected by spatial patterns at higher trophic levels (e.g., in the fish community).

### Survey data

Data were used on fish species composition (presence-absence) from the entire Barents Sea. Fish were sampled during the joint Russian-Norwegian ecosystem survey in August-September 2004–2012 by the Institute of Marine Research (IMR, Norway) and the Knipovich Polar Research Institute of Marine Fisheries and Oceanography (PINRO, Russia) using a demersal Campelen shrimp trawl (see [24] for a description of gear and survey design). More than 100 fish species were regularly caught during these ecosystem surveys. Pelagic species were removed from the dataset, as well as species that were present <3 times. The deletion of pelagic species was carried out because the fish were sampled using demersal trawls and would therefore not fully represent the full range of pelagic species present. Due to uncertainties regarding species identification, four taxa were only identified to genus level (*Ammodytes* spp. *Careproctus* spp., *Icelus* spp., *Liparis* spp.), whereas unidentified *Zoarchidae* species (i.e., those *Zoarchidae* species that are not easily identified to species level) were identified to the family level. Deep (>500 m) and shallow (<50 m) stations were excluded due to unrepresentative sampling. Our final data matrix included 3661 stations with presence-absence data for 60 fish taxa (Table 1).

**Table 1:** Overview of fish taxa assessed in this study.

Latin name	Common name
CHIMAREIDAE	** **
*Chimaera monstrosa*	Rabbit fish
RAJIDAE	
*Amblyraja hyperborea*	Arctic skate
*Amblyraja radiata*	Thorny skate
*Bathyraja spinicauda*	Spinetail ray
*Raja clavata*	Thornback ray
*Rajella fyllae*	Round ray
ARGENTINIDAE	
*Argentina silus*	Greater argentine
GADIDAE	
*Brosme brosme*	Tusk
*Enchelyopus cimbrius*	Four-bearded rockling
*Gadus morhua*	Atlantic cod
*Gadiculus argenteus*	Silvery pout
*Gaidropsarus argentatus*	Arctic rockling
*Melanogrammus aeglefinus*	Haddock
*Merlangius merlangus*	Whiting
*Micromesistius poutassou*	Blue whiting
*Molva molva*	Ling
*Pollachius pollachius*	Pollach
*Pollachius virens*	Saithe
*Trisopterus esmarkii*	Norway pout
MACROURIDAE	
*Macrourus berglax*	Onion-eye grenadier
ZOARCIDAE	
*Lycodes esmarkii*	Greater eelpout
*Lycodes gracilis*	Vahl’s eelpout
*Zoarcidae* spp.	Eelpouts
SCORPAENIDAE	
*Sebastes mentella*	Beaked redfish
*Sebastes norvegicus*	Golden redfish
*Sebastes viviparus*	Norway redfish
COTTIDAE	
*Artediellus atlanticus*	Atlantic hookear sculpin
*Artediellus scaber*	Hamecon
*Icelus* spp.	Scaled sculpins
*Gymnocanthus tricuspis*	Arctic staghorn sculpin
*Myoxocephalus scorpius*	Shorthorn sculpin
*Triglops murrayi*	Moustache sculpin
*Triglops nybelini*	Bigeye sculpin
*Triglops pingelii*	Ribbed sculpin
COTTINCULIDAE	
*Cottunculus microps*	Polar sculpin
AGONIDAE	
*Aspidophoroides olrikii*	Arctic alligatorfish
*Leptagonus decagonus*	Atlantic poacher
CYCLOPTERIDAE	
*Cyclopterus lumpus*	Lumpsucker
*Eumicrotremus derjugini*	Leatherfin lumpsucker
*Eumicrotremus spinosus*	Atlantic spiny lumpsucker
LIPARIDAE	
*Careproctus* spp.	Sea snails
*Liparis* spp.	Snailfishes
STICHAEIDAE	
*Anisarchus medius*	Stout eelblenny
*Leptoclinus maculatus*	Spotted snake blenny
*Lumpenus fabricii*	Slender eelblenny
*Lumpenus lampretaeformis*	Snake blenny
ANARHICHADIDAE	
*Anarhichas denticulatus*	Northern wolffish
*Anarhichas lupus*	Atlantic wolffish
*Anarhichas minor*	Spotted wolffish
PLEURONECTIDAE	
*Glyptocephalus cynoglossus*	Witch flounder
*Hippoglossoides platessoides*	Long rough dab
*Hippoglossus hippoglossus*	Halibut
*Limanda limanda*	Dab
*Microstomus kitt*	Lemon sole
*Pleuronectes platessa*	European plaice
*Reinhardtius hippoglossoides*	Greenland halibut
AMMODYTIDAE	
*Ammodytes* spp.	Sand lances
SYNGNATHIDAE	
*Entelurus aequoreus*	Snake pipefish
LOPHIIDAE	
*Lophius piscatorius*	Angler

In order to smooth out small-scale spatial variations in species composition, we aggregated the survey stations to larger grid cells. The complete survey area (the rectangular area defined by the maximum and minimum latitudes and longitudes of survey stations of the entire data set) was divided into 6 × 6 grid cells (the size of each cell was approximately 208 × 208 km). We thus ensured that each grid cell enclosed a sufficient (>5) number of sampling stations for each year. The number of grid cells per year varied between 24 and 28 as two of the grid cells were covered by land, whilst others were located outside the surveyed area in some or all of the years. In total 237 grid cells were assessed. Each species was given a biogeographic affiliation and classified as boreal, Arctic or arcto-boreal [24, 25].

### Functional trait matrix

The analyses were based on an updated version of a trait matrix provided by Wiedmann et al. [13]. The traits were carefully selected and interpreted in terms of the species’ ecosystem functions, concerning the flow and processing of energy and material [13]. The matrix consisted of 23 functional traits belonging to 8 trait categories (Table 2). The trait categories "feeding", "habitat", "offspring habitat", "offspring size", "body shape", "fecundity" and "body size" were obtained from the literature [13]. The trait category "environmental tolerance range" was calculated based on absolute values of temperature, salinity and depth ranges measured during the ecosystem surveys, independently of the species' actual preferences with regard to these environmental conditions. The environmental tolerance of fish species was coded as a categorical variable with three levels ("low tolerance range" ("1"), "medium tolerance range" ("2") and "high tolerance range ("3")).

**Table 2:** Overview of the functional traits used to calculate functional redundancy.

Trait	Trait category	Coding
Benthos feeder	Feeding	Binary [0,1]
Plankton feeder		
Fish feeder		
Benthos- and fish feeder		
Plankton- and fish feeder		
Demersal	Habitat	Binary [0,1]
Pelagic		
Demersal eggs	Offspring habitat	Binary [0,1]
Pelagic eggs		
Ovoviviparous		
Small (< 2 mm)	Offspring size	Binary [0,1]
Medium (2–8 mm)		
Large (> 8 mm)		
Normal	Body shape	Binary [0,1]
Flat		
Eel-like		
Elongated		
Deep and/or short		
Temperature range Salinity range Depth range	Environmental tolerance range	Ordinal [1,3] Temperature range: <4°C = "1"; 4–8°C = "2"; >8°C = "3" Salinity range: <1 = "1"; 1–2 = "2"; >2 = "3" Depth range: <200m = "1"; 200-400m = "2"; >400m = "3"
Mean fecundity	Fecundity	Continuous [counts]
Maximum body length	Body size	Continuous [cm]

### Calculating functional redundancy

**Fig 2:**
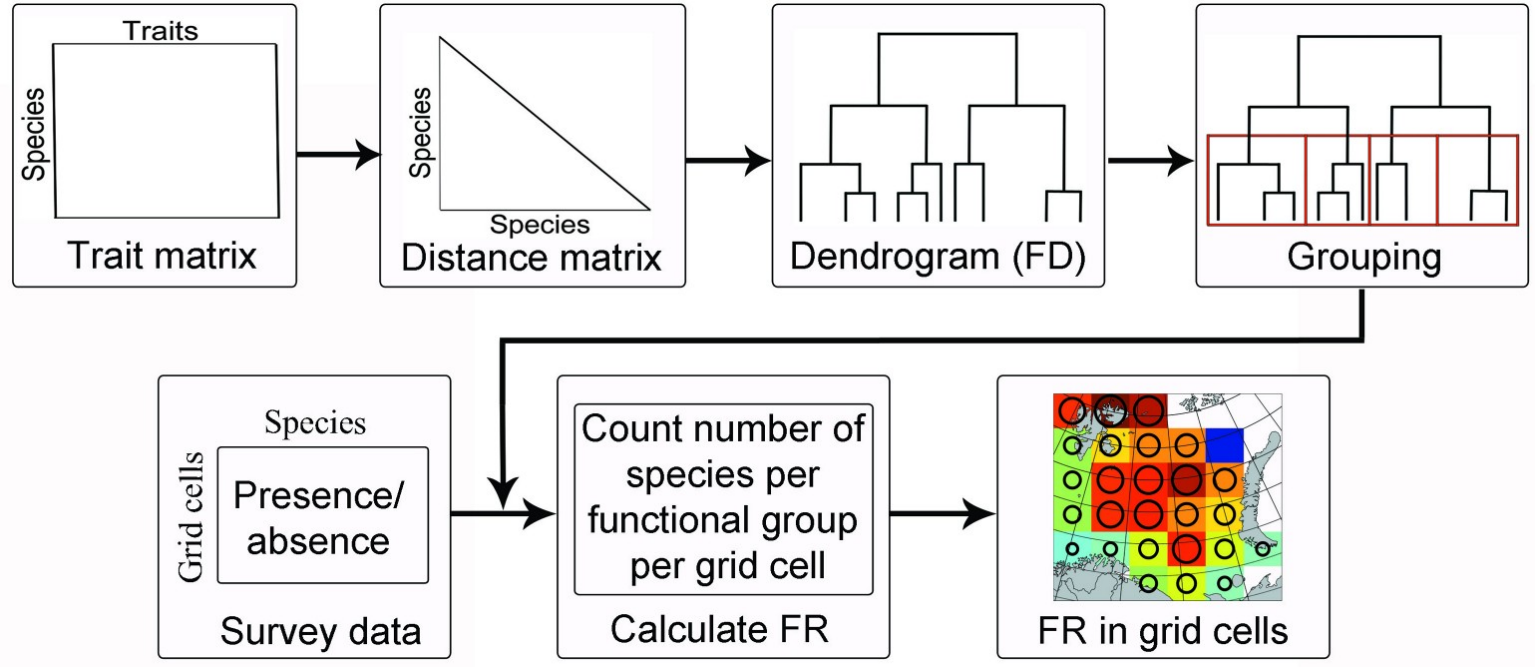
Procedure to calculate functional redundancy. See main text for details.

FR was computed as the number of species per functional group [8]. Calculating FR on the basis of presence/absence data (from the ecosystem survey) and the functional trait data, involved a four-step process (Fig 2).

1) Based on the functional characterization, the dissimilarities among species were calculated (i.e., how different the species are from one another with regard to their functional traits). The functional traits fall into three types of measurement scales: continuous, binary and ordinal (see Table 2). For mixed data Gower’s distance measure was used [26], including the enhancement for ordinal variables by Kaufman and Rousseeuw [27].

2) Based on the distance matrix, a dendrogram was constructed using the Ward clustering algorithm [28].

3) A permutation procedure described by Greenacre and Primicerio [29], designed to detect significant clustering of the species in the functional dendrogram, was used to determine the number of functional groups − for more details about this test see Greenacre [30].

4) Once the functional groups were identified, FR was estimated for a specific functional group in a grid cell (representing a year) and represented as the number of species members of that group found in the grid cell. To assess the proportion of functional groups present at the same time (i.e., in the same grid cell and in the same year), the degree of co-occurrence (DCO) was calculated as the FR in a grid cell in a given year divided by the number of taxa in that particular functional group. R software [31] and the following R libraries: StatMatch, vegan, splancs, rgdal, fields, gstat, FD, raster, maptools were used.

### Explanatory variables

Species' trophic interactions likely have significant impact on ecosystem dynamics and functioning [32]. In order to assess and compare the overall trophic status of the functional groups, indicative estimates of each species’ trophic level (TL) were extracted from FishBase [33]. In order to assess the temporal scales at which individuals from each functional group operate, estimates of each species' longevity were compiled based on literature reviews [14, 24, 33]. Additional ecological characteristics of the functional groups were assessed and compared by means of estimating maximum body length (ML) and environmental tolerance range of the species, both obtained from the trait matrix. The average values of TL, ML, environmental tolerance range and longevity were calculated for each functional group and compared to the groups using a simple ANOVA (α = 0.05). In cases where species were aggregated to a higher taxonomic level (e.g., *Zoarcidae* spp.), we calculated average values for the taxon based on values of the constituent species.

### Spatial and temporal dependence

In order to test for spatial and temporal dependence, counts of the functional groups for all the sample points (*n* = 237) were related to their corresponding grid cells and years. Discrete spatial and temporal explanatory variables, were defined using a canonical correspondence analysis (CCA). The significant contribution to the explained inertia of each explanatory variable was tested using a permutation test with 9999 permutations (R package vegan).

## Results

### Functional groups

**Fig 3:**
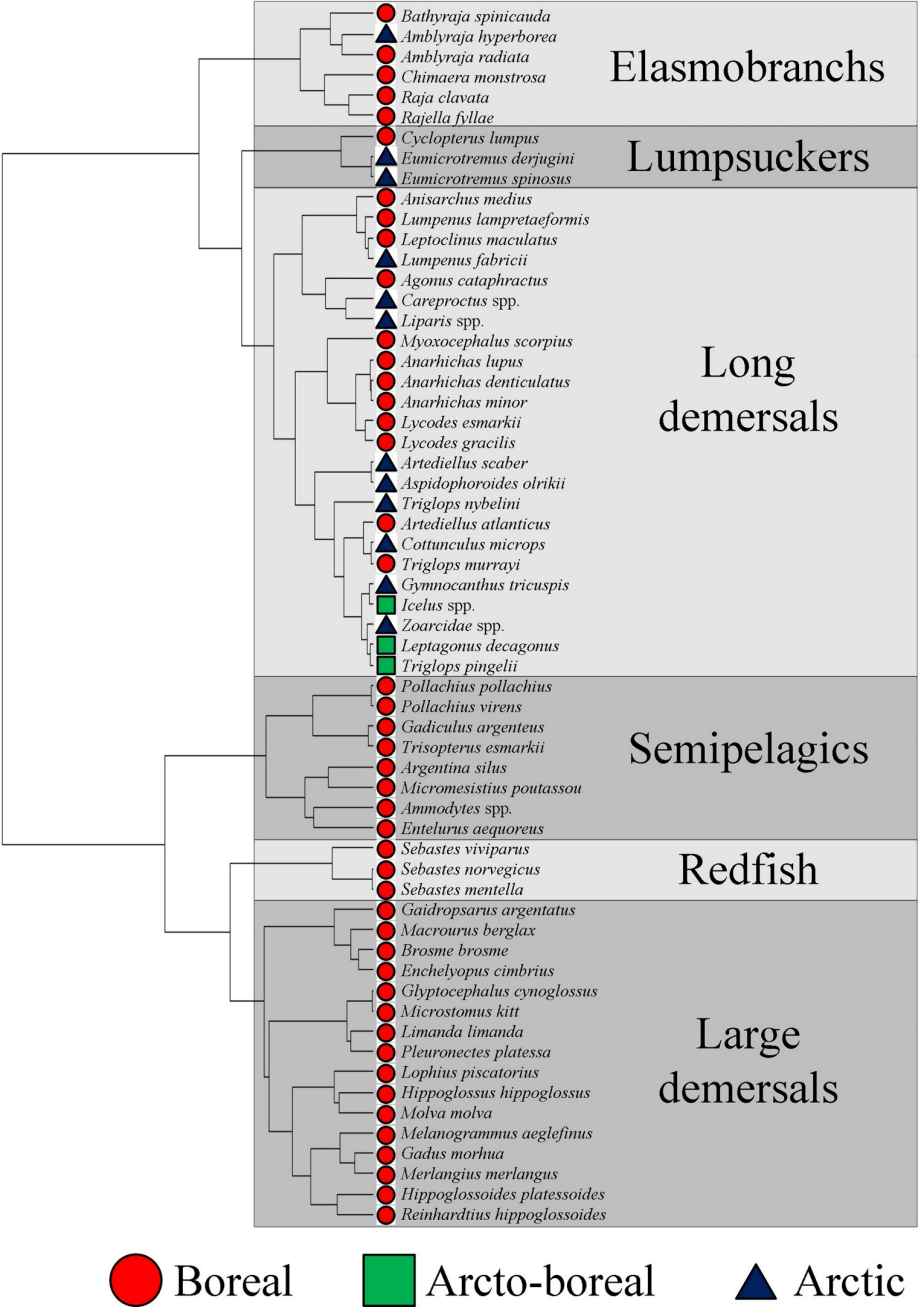
Functional dendrogram for 60 Barents Sea fish taxa, their biogeographical affiliations, and their functional groups.

The permutation procedure on the cluster dendrogram suggested a division of the species into six functional groups (*p* = 0.009). The groups were assigned short names on the basis of typical group characteristics (Fig 3). For instance, most of the members of the Long demersals group display an elongated or eel-like body shape, whereas the species of the Redfish group are all redfish belonging to the family *Sebastidae*. Although the groupings were based solely on the multivariate analysis of trait data, some groups consisted of phylogenetically closely related species (e.g., the redfish group), due to the effect of phylogenetic proximity on phenotypic similarity.

There were large spatial (between cells) differences across the Barents Sea in terms of functional group composition and smaller differences between the years. Of the total inertia in the samples-by-functional groups table of counts (a 273 × 6 matrix), 67.5% was explained by the spatial position and 3.7% by the years. Both these results, which quantify the level of spatial and temporal dependence respectively, are significant (permutation test, *p* < 0.0001).

The functional groups varied in the number of species (i.e., 3–24 taxa per functional group; Table 3) and with respect to biogeographic composition. The “redfish” and “lumpsuckers” groups, consisting of only three species each, were the smallest functional groups. The “long demersals” group, consisting of 24 taxa, including eelpouts (*Lycodes* spp.) and wolffish (*Anarhichas* spp.), constituted the most species-rich functional group and consisted of 12 boreal, three arcto-boreal and 9 Arctic species. The Elasmobranch group consisted of five boreal and one Arctic species, whereas the Lumpsuckers consisted of one boreal and two Arctic species. The three remaining functional groups consisted solely of boreal species.

**Table 3:** Summary of results. SD = standard deviation; FR = functional redundancy; DCO = degree of co-occurrence; ML = maximum body length (cm).

Group name	No of species	Biogeographic composition	Longevity in years (mean ± SD)	Trophic level (mean ± SD)	Body size (ML) (mean ± SD)	FR	Spatial coverage	DCO
Elasmobranch	6	5 boreal, 1 arctic	24 ± 14	3.9 ± 0.4	121 ± 39	Redundant in SW Declining redundancy towards NE	Expanding	Low
Lumpsuckers	3	1 boreal, 2 arctic	7.0 ± 6.9	3.6 ± 0.3	28 ± 28	Variable redundancy	Contracting until 2009, then small expansion	Low
Long demersals	24	12 boreal, 3 arcto-boreal, 9 arctic	12 ± 7.2	3.5 ± 0.2	45 ± 50	Redundant in central and N	Contracting until 2010, then small expansion	Medium
Semipelagics	8	All boreal	15 ± 11	3.6 ± 0.6	64 ± 44	Redundant in SW	Contracting	Low
Redfish	3	All boreal	58 ± 17	4.0 ± 0.06	64 ± 33	Redundant in SW Declining redundancy towards NE	Expanding	High
Large demersals	16	All boreal	25 ± 12	3.8 ± 0.4	127 ± 107	Redundant in SW Declining redundancy towards NE	No change	Low

There was significant variation in average longevity (F_5,54_ = 13.55, *p* < 0.001) among the functional groups (Table 3 and S1 Table). Across all taxa, the average longevity was 19 years (SD, 15 years). The Elasmobranch group had significantly higher longevity than the Long demersals. The Redfish had significantly higher longevity than all other groups. The Large demersals had significantly higher longevity than the Lumpsuckers, the Long demersals and the Semipelagics.

There was also significant variation in TL among the functional groups (F_5,54_ = 2.535, *p* = 0.0392) (Table 3 and S2 Table). Across all taxa, the average TL was 3.7 ± 0.4. The Long demersals had significantly lower TL than the Elasmobranch group, the Redfish and the Large demersals.

Finally, there was significant variation in ML among the functional groups (F_5,54_ = 3.565, *p* = 0.00739) (Table 3 and S3 Table). Across all taxa, the average ML was 78 ± 76 cm. The Elasmobranch group and the Large demersals displayed the higher average ML, whereas the Lumpsuckers and the Long demersals showed the lowest average ML (Table 3). The Elasmobranch group was significantly longer than the Lumpsuckers, the Long demersals and the Semipelagics, whereas the Large demersals were significantly longer than the Long demersals. No significant difference was observed among the functional groups (F_5,54_ = 0.806, *p* = 0.551) (S3 Table).

### Spatio-temporal patterns of functional redundancy

**Fig 4:**
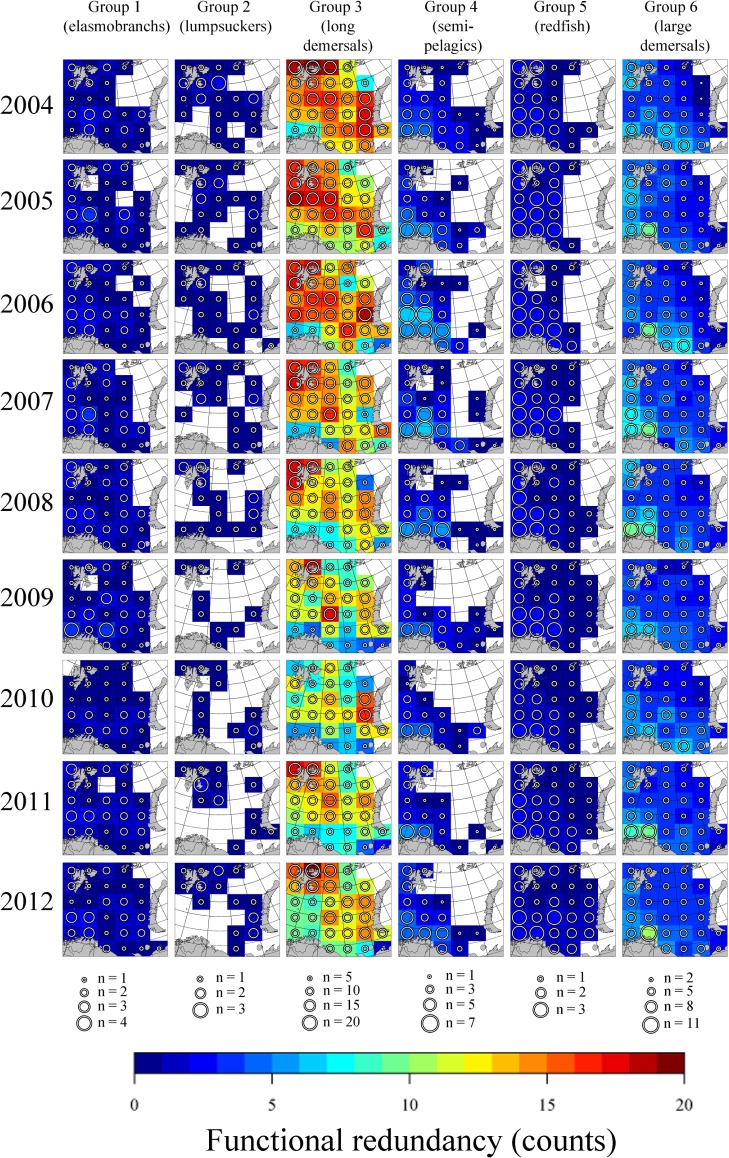
Functional redundancy of demersal fish in the Barents Sea in the period 2004–2012, presented by colour scale and circle size. For each grid cell, functional redundancy was measured as the number of species present per functional group. Circle size varies between the functional groups.

FR in the Barents Sea varied between areas and functional groups. Some functional groups displayed persistent spatial patterns (Fig 4); for instance, the Elasmobranchs, which primarily consisted of boreal species, were most redundant in the south-west, with decreasing redundancy towards the north-east. The Lumpsuckers, which consisted of a mix of Arctic and boreal species, displayed scattered and variable distributions in the central, northern and eastern Barents Sea. The Long demersals, where half of the species were boreal and the rest either Arctic or arcto-boreal, covered the entire Barents Sea. They were however, most redundant in the central and northern Barents Sea. The Semipelagics, which were boreal, were distributed in the south-west. The redfish and the large demersals, all of which were boreal, displayed the highest redundancy in the south-west and decreased redundancy towards the north-east.

**Fig 5:**
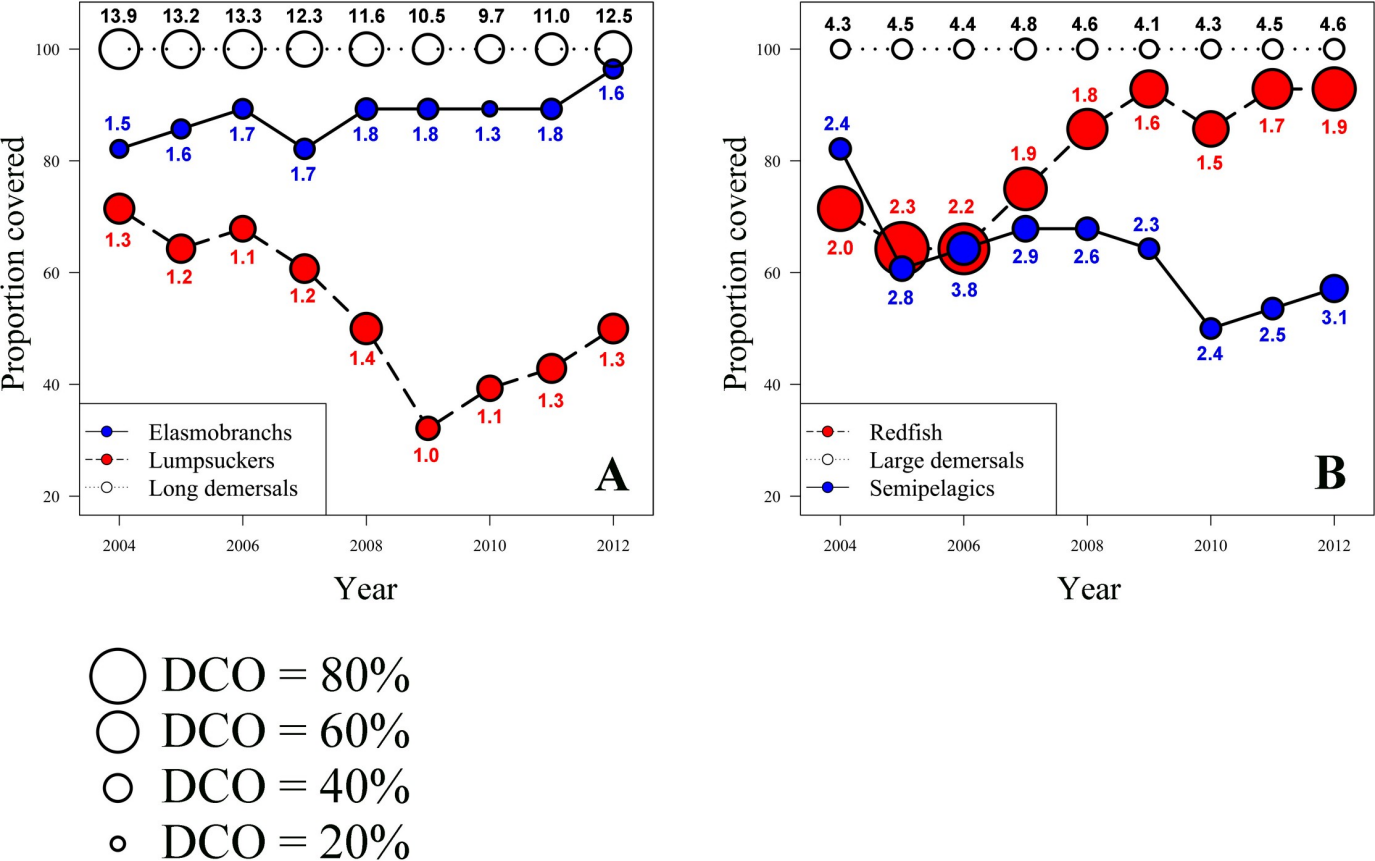
Proportion of the Barents Sea covered by the individual functional groups in the period 2004–2012, expressed as spatial coverage in %, for **(A)** Elasmobranchs, Lumpsuckers and Long demersals, and for **(B)** Redfish, Large demersals and Semipelagics. The numbers indicate the actual average functional redundancy for each functional group by year. Circle size indicates the degree of co-occurrence (DCO), i.e. for each functional group and each year, the actual average functional redundancy was divided by the number of taxa in that group.

Five patterns of spatio-temporal variation in FR were prominent (Fig 5). Elasmobranchs gradually expanded their distribution area in the study period (*R*^2^ = 0.56, *p* = 0.01), from ~80% in 2004 to almost 100% in 2012, showing a rather stable average FR of 1.3–1.8, which corresponds to a low DCO of ~20–30%. Lumpsuckers’ distribution showed a contraction (*R*^2^ = 0.57, *p* = 0.01), from >70% coverage in 2004 to ~30% coverage in 2009, and a subsequent expansion to ~45% coverage in 2012. The Long demersals displayed an overall decline in the average FR (*R*^2^ = 0.40, *p* = 0.04), from 13.9 in 2004 to 9.7 in 2010, and a subsequent increase back to 12.5 in 2012 (Fig 5). The distribution of the Semipelagics showed a contraction (*R*^2^ = 0.49, *p* = 0.02), from ~80% coverage in 2004 to ~50–60% in 2010–2012. Finally the Redfish, which showed high DCO values of ~50–80%, expanded their distribution (*R*^2^ = 0.75, *p* < 0.002), from ~65% coverage in 2004 to >90% coverage in 2012. The data indicated that the expansion was largely due to one species (*Sebastes mentella*) and therefore a decline in the average FR was evident (*R*^2^ = 0.38, *p* < 0.05). There was no evident pattern in spatio-temporal variation in FR for the Large demersals.

## Discussion

### Functional groups

Any system may become vulnerable if ecologically relevant functional groups have low redundancy. For instance, in the species rich (>3000 species) Great Barrier Reef ecosystem, one parrotfish species (*Bolbometopon muricatum*) was the main consumer of reef carbonates. In regions where fishing reduced this species severely, the reefs shifted from steady-state calcification towards carbonate accumulation [34]. Mapping of FR is therefore a simple and transparent way to assess the sensitivity of an ecosystem to species loss.

The analyses suggest that six functional groups of demersal fish play contrasting functional roles in the Barents Sea ecosystem. Although the species within each functional group are not functionally identical, there is less intragroup than intergroup variation in trait values. The species that make up a particular functional group have many functional and life history traits in common. A significant intergroup variation in longevity and TL values was observed, and although none of the functional traits that were used inform explicitly about the biogeographic affiliations of each species, a clear intergroup variation in such affiliation was evident. In fact, the functional dendrogram (Fig 3) consisted of two main branches and all the Arctic and arcto-boreal taxa occured in the uppermost branch, and all but one of them occurred either in the Long demersals or the Lumpsuckers. This suggests that an Arctic affiliation requires particular functional adaptation, as supported by a recent study which showed that typically Barents Sea Arctic fish species are small-sized, bottom-dwelling and benthivorous [35].

The species occurring in a particular functional group represent a unique combination of functional trait values, which discriminates them from species belonging to the other functional groups. For instance, the Long demersals group consists mostly of small, relatively short-lived, demersal, benthos-feeders found at below-average trophic levels. Half of the species in this group were either Arctic or arcto-boreal. The Long demersals are reported to be largely non-migratory [24]. They are therefore likely proficient at utilizing the local benthic resources including small-sized food items. An elongated or eel-like shape may facilitate digging into the sediment for prey. Some of the Long demersals (e.g., the elongated *Artediellus atlanticus* and *Gymnocanthus tricuspis*) are reported to prey on burrowing animals, whereas others (including species of the eel-like genera *Lumpenus* and *Lycodes*) are known to dig holes into sediment [24]. Although the European eel (*Anguilla Anguilla*) conduct a spawning migration across the Atlantic Ocean from Europe to the Sargasso Sea [36], the eel-like shape is not ideal for long migrations. This partly explains the non-migratory behaviour of many of the Long demersals. Apart from the abundant polar cod (*Boreogadus saida*) and a few species of low abundance (e.g., Arctic cod *Arctogadus glacialis*, navaga *Eleginus nawaga* and Arctic flounder *Liopsetta glacialis*) none of the truly Arctic fish species are migratory [37]. There is therefore very little horizontal energy flux in Arctic fish communities. Species from the Long demersals group have been recorded in the stomachs of a wide range of predators, including seabirds, seals and elasmobranchs, as well as large bodied, teleost fish species including long rough dab (*Hippoglossoides platessoides*), Greenland halibut (*Reinhardtius hippoglossoides*) and Atlantic cod [33, 38]. This suggests that the Long demersals play an important functional role in the ecosystem by converting energy and matter from the benthos (including endobenthos) to species at higher TL.

The Elasmobranchs are demersal generalists found at above-average trophic levels, as they prey on benthos, fish and in some instances dead organic material (i.e., they are scavengers; [39]). Most species members of the Elasmobranch group have a flat body shape, a property they share with the flatfish from the Large demersals group. Fish with a flat body shape have a negative buoyancy [40], which naturally promotes a benthic affiliation. Flat shaped fish species may glide efficiently above the seabed [41], but since they need to spend energy in order to move up and stay above the seabed [40], the flat shape does not encourage long migrations. A recent tagging experiment carried out in British waters revealed that the common skate, *Dipturus batis*, a close taxonomic relative, showed a strong site fidelity [42]. Although the rabbit fish, *Chimaera monstrosa*, conducts spawning migrations during summer time [24], their traits suggest that they are mostly stationary and proficient at exploiting local resources. The elasmobranchs are generally large and long-lived, which suggests that they store large amounts of energy over long periods of time and therefore are a predictable ecosystem component. They do however have low resilience to fishing and other sources of increased mortality [43]. Several of the Elasmobranchs are scavengers [38, 44, 45], a property that they presumably benefit from in the Barents Sea where commercial fishing provides discards. This opportunistic feeding behaviour, combined with a low number of natural enemies, suggests that this group is an important structural ecosystem component [46, 47].

The Lumpsucker group consists of three round-bodied plankton feeders occurring at below-average trophic levels. One of the species (*Cyclopterus lumpus*) is boreal, semipelagic, eurybatic and up to 50 cm long, whereas the two other species (*Eumicrotremus spinosus* and *E*. *derjugini*) are Arctic, demersal, shallow-water and less than 14 cm long [24]. The latter are among the few plankton feeding Arctic fish found in the Barents Sea. Contrary to what one would expect from the body shape, *C*. *lumpus* is a good swimmer, performing extensive migrations between the open ocean (where it feeds) and coastal areas (where it reproduces) [48, 49]. *Cyclopterus lumpus* is one of the few species that utilize comb jellies (*Ctenophora*) as food [38, 50]. This may be an important contribution to the overall ecosystem functioning as ctenophores are expected to become more abundant with climate warming [51]. The two Arctic lumpsuckers are slow swimmers and feed on hyperiids [38, 52], they have no major seasonal migrations, but contribute to local communities throughout the year. *C*. *lumpus* is eaten by Greenland shark, Atlantic wolfish *Anarhichas lupus*, Greenland halibut, sea birds, seals, whales and other mammals [53], *E*. *spinosus* has been observed in the stomachs of elasmobranchs (e.g., *Amblyraja radiata*; [54]), Atlantic cod (*Gadus morhua*) and spotted wolffish (*Anarhichas minor*) [38]. The Lumpsuckers therefore provide a small but important functional group, linking planktonic species to large members of several other functional groups, in both Atlantic and Arctic waters in the Barents Sea.

The Semipelagics consisted of boreal, pelagic-demersal species, and they comprised the species with a certain degree of pelagic affinity, the purely pelagic species (capelin *Mallotus villosus*, Atlantic herring *Clupea harengus* and polar cod *Boreogadus saida*) being excluded from our analyses. The Semipelagics are plankton feeders found at below-average trophic levels. They use both the pelagic and demersal habitats, and although many are schooling species they commonly do not perform long migrations. Nevertheless, since the Semipelagics are, in general, streamlined in shape, they are good swimmers and have a relatively large spatial range. Unlike the adults of many of the other fish species in the Barents Sea (except for a number of strictly pelagic and highly migratory species not included in the present analyses), they prey on small food items such as zooplankton (both benthic and pelagic), euphausiids and fish eggs and larvae [33, 38]. As prey species, they transfer this energy to larger predators such whales, seals, sea birds, squids, elasmobranchs and large number of teleost fish which mostly belong to the Large demersals group [33, 38]. In contrast to the long demersals, which transfer energy from the benthos towards higher TL, the Semipelagics play a role as foraging species, preying on plankton and being important food sources for predators at higher TL.

The Redfish group consisted of boreal, benthopelagic, normally (fusiform) shaped and relatively large species, all belonging to the family *Sebastidae*. They are found at above-average trophic levels, and have the highest average longevity among the functional groups. Redfish move between the bottom and the pelagic water layers, and the two largest members of the group (i.e., *S*. *mentella* and *S*. *norvegicus*) perform extensive migrations [55, 56]. Redfish prey on smaller (mostly pelagic) fish, zoobenthos and large zooplankton (e.g., shrimps and euphausiids). They have been found in the stomachs of various elasmobranchs and also teleost fish belonging to various functional groups, including long rough dab, Northern wolfish (*Anarhichas denticulatus*), Arctic rockling (*Gaidropsarus argentatus*), blue whiting (*Micromesistius poutassou*), haddock (*Melanogrammus aeglefinus*), Greenland halibut and Atlantic cod [33, 38]. Redfish are therefore important members of the Barents Sea food web as they interact with and connect many other ecosystem components, including large crustaceans and several functional groups of fish [17, 33, 56]. Furthermore, due to their high longevity, they provide this ecosystem function over a long time span, and their presence represents a stable ecosystem component.

As their name implies, the Large demersals are relatively large, demersal species found at above-average trophic levels. They feed on a variety of benthos and fish species [33]. They are all boreal, and all produce high numbers of pelagic eggs. Their potential to distribute their offspring across large areas is therefore high in comparison to members of other functional groups. Although some of the species in this group are apparently non-migratory (e.g., the roughhead grenadier, *Macrourus berglax*), most of them are migratory, with good swimming capabilities. Furthermore, the Large demersals are primarily omnivorous [35], and many of them are found across a much larger latitudinal range than for example members of the Long demersals. The Atlantic cod for instance is found across most shelf areas of the North Atlantic [33], whereas many of the Long demersals and Lumpsuckers are restricted to limited areas of the Barents Sea [57]. It can therefore be presumed they can respond quickly to environmental change [58]. Large demersals prey on many species, and as adults have, in general, few predators [33], which is indicative of high trophic levels. Recent food web analyses confirm that several of the Large demersals (e.g., cod and haddock) play very important roles as connectors both within and between food web compartments in the Barents Sea [17].

### Persistent patterns of functional redundancy

In this study, we observed several persistent, large-scale patterns of FR in Barents Sea fish. In the following, we argue that these patterns reflect the species' adaptations to the spatially differing environmental conditions. The central Barents Sea (Polar Front area), where warm and saline Atlantic water meets cold and less saline Arctic water, marks the northern limit of boreal species and the southern limit of Arctic species. Water temperature positively correlates with the metabolic rate of a species and therefore its energy demands [59]. It can therefore be assumed that fish residing in the cold, north-eastern part of the Barents Sea study area in general have lower energy demands than those residing in the warmer part.

In addition to contrasting water mass characteristics, Atlantic and Arctic waters differ when it comes to the prevailing carbon flux regime. In Atlantic water, the annual estimated gross primary production and vertical export of carbon is higher than in Arctic water masses. The proportion of carbon being exported towards the bottom is however higher in Arctic than in Atlantic water masses [23]. A high overall production and vertical export in the south-west apparently supports a high demersal and pelagic diversity, which in turn is reflected by a relatively high redundancy in four out of six functional groups: the Elasmobranchs, the Semipelagics, the Redfish and the Large demersals. In the Arctic, relatively low primary production rates, combined with high rates of vertical carbon flux, leaves relatively small amounts of energy in the upper water levels. This in turn may explain the low diversity of planktivorous species [5]. The Arctic carbon export regime thus favours a benthic affiliation, which is supported by the fact that the functional groups most common in the Arctic (e.g., the Long demersals) are dominated by bottom-dwelling, benthivorous species [35]. Yet, the amounts of carbon reaching the seabed in the Arctic part of the Barents Sea is limited, with high seasonal and inter-annual variability [23]. This calls for additional adaptations in order to reduce energy demands of the species living there. For instance, metabolic rate is also positively correlated with body mass [59]. The two functional groups that comprised most of the cold-water adapted, Arctic fish species (i.e., the Lumpsuckers and the Long demersals) also displayed the smallest average body sizes, thus further reducing their energy demands.

Body shape is another cold-water adaptation with implications for metabolic rate [59]. Fish found in Atlantic water masses of the Barents Sea display a variety of body shapes, including fusiform, flat, deep/short, elongated and eel-like shapes, whereas those found in Arctic waters are typically elongated or eel-like. Codfish (order Gadiformes), a dominating group of fish in the central and Atlantic parts of the Barents Sea, have a fusiform body shape, which is shown to have a high resting metabolic rates [59]. Eelpouts, a specious an eel-like shaped family common in the central and Arctic parts of the Barents Sea, typically display low resting metabolic rates [59]. Typically fish species from Arctic waters in the Barents Sea have body shapes that promote low resting metabolic rates. Furthermore, at low swimming speeds, evidence suggests that an eel-like shape is more power efficient than a fusiform shape [60], indicating limited motion, modest swimming speeds and a non-migratory behaviour. In the case of avoidance, fish species with eel-like body shape are however able to escape explosively (personal observation).

### Spatio-temporal variation in functional redundancy

The study period 2004–2012 was characterized by a warming of the water masses in the Barents Sea, expressed by the expansion of warm Atlantic and mixed water masses and retreating cold Arctic water masses [15, 16]. This enabled a north-eastward movement of large, boreal, omnivorous fish species, replacing small-sized Arctic benthivorous fish (i.e., *borealization*) [12, 14, 17, 35]. Consequently, the functional groups developed differently in space during the study period. Several of the functional groups that consisted of mainly boreal species increased their redundancy in the northeast. This is supported with the results from a recent study showing that fish species having a strong swimming capacity, omnivorous diet and a large latitudinal range have a high capacity to redistribute in response to environmental change [58]. Fast-swimming fish include species that migrate pelagically [61], and in the last two decades a reduction in sea ice concentration and an increase in seawater temperature in the Bering Sea coincided with a shift from an ecosystem characterized by tight benthic-pelagic coupling to a pelagically dominated one [62]. We focus on demersal fish species, large, long-lived, migratory, demersal predators, including cod, haddock, long rough dab and beaked redfish typically carried out the borealization of traditionally Arctic areas of the Barents Sea [12]. In contrast to the typical Arctic fish species [35], most of these demersal, boreal species that move into the Arctic have a certain pelagic affiliation, as they have some pelagic species in their diet [24]. A recent study showed that boreal species that move into the Arctic have a more generalistic feeding pattern than the Arctic ones [17]. This indicates that the ongoing borealization promotes a higher degree of both vertical and horizontal energy flux in the former Arctic parts of the Barents Sea.

In contrast to the functional groups consisting of boreal species, the functional groups consisting of primarily Arctic species became less redundant in time and space, a pattern that was particularly prominent in the period 2004–2010. For instance, the Long demersals, which was the most species rich functional group and displayed the highest redundancy, underwent a reduction in FR throughout the Barents Sea. Although this group remained redundant in most parts, the reduction nearly caused a total loss of redundancy in the southeast, partly explaining a previous reduction in FD [13]. In the period 2004–2012, the area covered by Arctic fish communities in the Barents Sea diminished and became increasingly more restricted to the north-easternmost parts [12]. A further northward retraction of true Arctic species is most likely restricted by the extent of the shallow Barents Sea shelf area. The environmental conditions in the Arctic Central Basin appear to be inappropriate for most fish, as only 13 fish species have been recorded there [5]. This may be due to the greater depths, or because the nutrient level is insufficient in these almost permanently ice-covered areas [63]. Several other Arctic shelf seas however still display Arctic environmental conditions. For instance, the adjacent Kara Sea is cold [64] and ice covered most of the year [65]. Indeed, many of the Arctic fish species found in the Barents Sea are also present in the Kara Sea [66], and may continue to reside there if the appropriate environmental conditions persist.

The apparent decline in the Lumpsucker redundancy was probably partly caused by the fact that the most widespread of the group members (lumpsucker, *C*. *lumpus*) showed a strong biomass reduction in the Barents Sea in the period 2007–2010 [67]. This species is also mostly found in the pelagic zone in the autumn when the Joint Norwegian-Russian ecosystem survey is conducted [24], resulting in relatively low catchability when using demersal trawl gear. The two other group members (*E*. *derjugini* and *E*. *spinosus*), both preferring cold waters, are only found on shallow banks in the north and east, and may therefore encounter difficulties if the water masses at the banks heat up. The future state of the small Lumpsucker group requires further attention, as the group possesses a specific role in the ecosystem which, if lost, may reduce the amount energy from sinking organic matter that is fed directly back into the higher trophic levels.

### Stressors and vulnerability

Although the functional group approach allows general, large-scale species composition patterns to be addressed, it tends to mask abundance-related ecological features. Although spatio-temporal patterns in FR appeared to be relatively stable, the cod abundance has rapidly increased in the northern Barents Sea in the later years as a response to a warmer climate [68, 69]. A similar pattern could be recognized in the Large demersals group, where long rough dab currently display a northward distribution shift [12]. However, such changes may have implications beyond purely spatial ones. Intensified fisheries in the northern Barents Sea is one expected consequence of the northward shift of boreal, commercially valuable species. Fisheries impact species across many functional groups, not only target species, and as such, future Arctic fish communities (e.g., the Long demersals) may need to cope with intensified fishery-induced disturbance. On the other hand, the small (three species) Redfish group displayed a spatial expansion during the study period, to cover the entire Barents Sea in 2012, despite one of the group members (the golden redfish, *Sebastes norvegicus*) presently being at a historically low abundance level [70]. Redfish share several life history traits that make them sensitive to fishing, exemplified by large body size, high longevity and late maturation [14]. Caution is thus required to avoid decimation of this small predatory group of species.

## Conclusions

In this study, we show that the redundancy of functional groups of fish in the Barents Sea varied in space and time during 2004–2012. Fish communities in the Arctic part of the Barents Sea have different functional configurations than those boreal communities in Atlantic water masses. On the other hand, the central Barents Sea appeared to sustain diverse demersal assemblages including both Arctic and boreal species. Persistent spatial patterns in FR may therefore largely reflect adaptations to spatial variations in water mass characteristics and vertical carbon flux in the Barents Sea. However, recent environmental change has caused the water masses in the Barents Sea to heat up and the sea ice to retreat. Consequently, a borealization of Arctic fish communities has occurred in which boreal fish have moved north-eastwards into previously Arctic areas. Typical Arctic species have been marginalized in the Barents Sea. The borealization of the fish community implies an introduction of typical boreal functional traits to the Arctic environment, as well as a diminishing representation of typical Arctic traits. Such borealization will also lead to an altered FR in the Barents Sea. The functional groups dominated by boreal species generally increased their redundancy in the central and north-eastern Barents Sea whilst those groups that were dominated by Arctic species showed a decline in redundancy values which calls for further study. Furthermore, there are several speciose functional groups of fish in the Barents Sea, some groups are species poor and are hardly able to buffer against species loss. Thus, the changes in FR of fish communities may affect the future ecosystem functioning in the Barents Sea. Functional redundancy is a transparent metric that may help inform management on ecosystem functioning and vulnerability.

## Supporting information

S1 Table Summary statistics of ANOVA on the species' longevity as function of functional group. Significant (alpha = 0.05; Bonferroni corrected alpha = 0.005) relationships in longevity among functional groups are indicated in bold.
(DOCX)

S2 Table Summary statistics of ANOVA on the species' trophic level (TL) as function of functional group. Significant (alpha = 0.05; Bonferroni corrected alpha = 0.005) relationships in longevity among functional groups are indicated in bold.
(DOCX)

S3 Table Summary statistics of ANOVA on the species' maximum body length (ML) as function of functional group. Significant (alpha = 0.05; Bonferroni corrected alpha = 0.005) relationships in longevity among functional groups are indicated in bold.
(DOCX)

## References

[1] Poloczanska ES, Brown CJ, Sydeman WJ, Kiessling W, Schoeman DS, Moore PJ, et al Global imprint of climate change on marine life. Nature Clim Change. 2013; 10.1038/nclimate1958

[2] Larsen JN, Anisimov OA, Constable A, Hollowed AB, Maynard N, Prestrud P, et al editors. Climate Change 2014: Impacts, Adaptation, and Vulnerability. Part B: Regional Aspects Contribution of Working Group II to the Fifth Assessment Report of the Intergovernmental Panel on Climate Change. New York and Cambridge: Cambridge University Press; 2014 pp. 1567–1612.

[3] Overland JE, Stabeno PJ. Is the climate of the Bering Sea warming and affecting the ecosystem? Eos. 2004; 85: 309–316.

[4] Kortsch S, Primicerio R, Beuchel F, Renaud PE, Rodrigues J, Lønne OJ, et al Climate-driven regime shifts in Arctic marine benthos. Proc Natl Acad Sci USA. 2012; 109: 14052–14057. 10.1073/pnas.1207509109 22891319 10.1073/pnas.1207509109 PMC3435174

[5] Christiansen JS, Reist JD. Fishes In: Meltofte H, editor. Arctic Biodiversity Assessment. Conservation of Arctic Flora and Fauna (CASS). Akureyri: Narayana Press; 2013 pp. 192–245.

[6] Petchey OL, Gaston KJ. Functional diversity (FD), species richness and community composition. Ecol Lett. 2002; 5: 402–411.

[7] Laliberté E, Legendre P. A distance-based framework for measuring functional diversity from multiple traits. Ecology. 2010; 91: 299–305. 20380219 10.1890/08-2244.1

[8] Laliberté E, Wells JA, DeClerck F, Metcalfe DJ, Catterall CP, Queiroz C, et al Land-use intensification reduces functional redundancy and response diversity in plant communities. Ecol Lett. 2010; 13: 76–86. 10.1111/j.1461-0248.2009.01403.x 19917052 10.1111/j.1461-0248.2009.01403.x

[9] Levin SA, Lubchenco J. Resilience, robustness, and marine ecosystem-based management. BioScience. 2008; 58: 27–32.

[10] Ehrlich P, Walker B. Rivets and redundancy. BioScience. 1998; 48: 387.

[11] Palumbi SR, McLeod KL, Grünbaum D. Ecosystems in action: lessons from marine ecology about recovery, resistance, and reversibility. BioScience. 2008; 58: 33–42.

[12] Fossheim M, Primicerio R, Johannesen E, Ingvaldsen R, Aschan M, Dolgov A. Recent warming leads to a rapid borealization of fish communities in the Arctic. Nat Clim Change. 2015; 5: 673–677.

[13] Wiedmann MA, Aschan M, Certain G, Dolgov A, Greenacre M, Johannesen E, et al Functional diversity of the Barents Sea fish community. Mar Ecol Prog Ser. 2014; 495: 205–218.

[14] Wiedmann MA, Primicerio R, Dolgov A, Ottesen C, Aschan M. Life history variation in Barents Sea fish: implications for sensitivity to fishing in a changing environment. Ecol Evol. 2014; 4: 3596–3611. 10.1002/ece3.1203 25478151 10.1002/ece3.1203 PMC4224534

[15] Johannesen E, Ingvaldsen RB, Bogstad B, Dalpadado P, Eriksen E, Gjøsæter H, et al Changes in Barents Sea ecosystem state, 1970–2009: climate fluctuations, human impact, and trophic interactions. ICES J Mar Sci. 2012; 69: 880–889.

[16] Smedsrud LH, Esau I, Ingvaldsen RB, Eldevik T, Haugan PM, Li C, et al The role of the Barents Sea in the Arctic climate system. Rev Geophys. 2013; 51: 415–449.

[17] Kortsch S, Primicerio R, Fossheim M, Dolgov AV, Aschan M. Climate change alters the structure of arctic marine food webs due to poleward shifts of boreal generalists. Proc R Soc Lond B Biol Sci. 2015; 282: 10.1098/rspb.2015.1546 26336179 10.1098/rspb.2015.1546 PMC4571709

[18] Ozhigin VK, Ingvaldsen RB, Loeng H, Boitsov VD, Karsakov AL. The Barents Sea In: Jacobsen T, Ozhigin VK, editors. The Barents Sea. Ecosystem, resources, management. Half a century of Russian-Norwegian cooperation. Trondheim: Tapir Academic Press; 2011 Pp. 39–76.

[19] Loeng H. Features of the physical oceanographic conditions of the Barents Sea. Polar Res. 1991; 10: 5–18.

[20] Oziel L, Sirven J, Gascard JC. The Barents Sea frontal zones and water masses variability (1980–2011). Ocean Sci. 2016; 12: 169–184.

[21] Lien VS, Vikebø F, Skagseth Ø. One mechanism contributing to co-variability of the Atlantic inflow branches to the Arctic. Nat Commun. 2012; 4: 1488. 10.1038/ncomms2505 PMC3586715 23403588

[22] Overland JE, Wang M. When will the summer Arctic be nearly ice free? Geophys Res Lett. 2013; 40: 2097–2101.

[23] Reigstad M, Carroll J, Slagstad D, Ellingsen I, Wassmann P. Intra-regional comparison of productivity, carbon flux and ecosystem composition within the northern Barents Sea. Prog Oceanogr. 2011; 90: 33–46.

[24] Wienerroither R, Johannesen E, Dolgov A, Byrkjedal I, Bjelland O, Drevetnyak K, et al. Atlas of the Barents Sea Fishes. IMR/PINRO Joint Report Series. 2011; No 1/2011.

[25] Andriyashev AP, Chernova NV. Annotated list of fishlike vertebrates and fish of the Arctic seas and adjacent waters. J Ichthyol. 1995; 35: 81–123.

[26] Gower JC. A general coefficient of similarity and some of its properties. Biometrics. 1971; 27: 857−874.

[27] Kaufman L, Rousseeuw PJ. Finding Groups in Data: An Introduction to Cluster Analysis. New York: Wiley; 1990.

[28] Ward JH. Hierarchical grouping to optimize an objective function. J Am Stat Assoc. 1963; 58: 236–244.

[29] Greenacre M, Primicerio R. Multivariate Analysis of Ecological Data. Bilbao: Fundación BBVA; 2013. 336 pp.

[30] Greenacre M. A simple permutation test for clusteredness. Working Paper 1271. Department of Economics and Business, Universitat Pompeu Fabra. 2011. https://econ-papers.upf.edu/ca/paper.php?id=1271.

[31] R Development Core Team (2015). R: A Language and Environment for Statistical Computing. Vienna, Austria: R Foundation for Statistical Computing.

[32] Thébault E, Loreau M. Trophic interactions and the relationship between species diversity and ecosystem stability. Am. Nat. 2005; 166: E95–E114. 10.1086/444403 16224699 10.1086/444403

[33] Froese R, Pauly D. FishBase; 2016 World Wide Web electronic publication www.fishbase.org.

[34] Bellwood DR, Hoey AS, Choat JH. Limited functional redundancy in high diversity systems: resilience and ecosystem function on coral reefs. Ecol Lett. 2003; 6: 281–285.

[35] Frainer A, Primicerio R, Kortsch S, Aune M, Dolgov AV, Fossheim M, et al Climate-driven changes in functional biogeography of Arctic marine fish communities. Proc Natl Acad Sci USA. 2017; 114, 12202–12207. 10.1073/pnas.1706080114 29087943 10.1073/pnas.1706080114 PMC5699037

[36] Tesch F.W. (Thorpe J.E., ed.). The eel, 5th ed Blackwell Science, Oxford, England 2993; 408 pp.

[37] Ponomarenko VP. Some data on the distribution and migrations of polar cod in the seas of the Soviet Arctic. Rapp p-v réun—Cons int explor mer. 1968; 158:131–135.

[38] Dolgov AV. Composition, formation and trophic structure of the Barents Sea fish communities. Murmansk: PINRO Press; 2016. 336 pp. (in Russian).

[39] Dolgov AV. Feeding and food consumption by the Barents Sea skates. J Northw Atl Fish Sci. 2005; 35: 495–503.

[40] Phleger CF. Buoyancy in marine fishes: direct and indirect role of lipids. Amer Zool. 1998; 38: 321–330.

[41] Takagi T, Kawabe R, Yoshino H, Naito Y. Functional morphology of the flounder allows stable and efficient gliding: an integrated analysis of swimming behavior. Aquat Biol. 2010; 9: 149–153.

[42] Wearmouth VJ, Sims DW. Movement and behaviour patterns of the critically endangered common skate *Dipturus batis* revealed by electronic tagging. J Exp Mar Biol Ecol. 2009; 380: 77–87.

[43] Dulvy NK, Reynolds JD. Predicting extinction vulnerability in skates. Conserv Biol. 2002; 16: 440–450.

[44] Dolgov AV, Drevetnyak KV, Gusev EV. The status of skate stocks in the Barents Sea. J Northw Atl Fish Sci. 2005; 35: 249–260.

[45] Dolgov AV, Johannesen E, Høynes Å. Fish. Main species and ecological importance In: Jacobsen T, Ozhigin VK, editors. The Barents Sea. Ecosystem, resources, management. Half a century of Russian-Norwegian cooperation, Trondheim: Tapir Academic Press; 2011 pp. 193–200.

[46] Ebert DA, Bizzarro JJ. Standardized diet compositions and trophic levels of skates (Chondrichthyes: Rajiformes: Rajoidei). Environ Biol Fish. 2007; 80: 221–237.

[47] Bornatowski H, Navia AF, Braga RR, Abilhoa V, Corrêa MFM. Ecological importance of sharks and rays in a structural foodweb analysis in southern Brazil. ICES J Mar Sci. 2014; 10.1093/icesjms/fsu025

[48] Rusyaev SM. On the spatial relationship between the spawning and feeding parts of the range of lumpfish *Cyclopterus lumpus* (Cyclopteridae) in the Barents Sea and adjacent waters (according to results of analysis of the size composition similarity). J Ichthyol. 2013; 53: 397–403.

[49] Kennedy J, Jónsson SÞ, Kasper JM, Ólafsson HG. Movements of female lumpfish (*Cyclopterus lumpus*) around Iceland. ICES J Mar Sci. 2014; 10.1093/icesjms/fsu170

[50] Kudryavtseva OYu. Lumpsucker of the Barents Sea and adjacent waters. Moscow: Nauka Publishing; 2008. 164 pp. (in Russian).

[51] Eriksen E, Bogstad B, Dolgov A, Beck IM (2018) Cod diet as an indicator of Ctenophora abundance dynamics in the Barents Sea. Mar Ecol Prog Ser. 2018; 591: 87–100.

[52] Berge J, Nahrgang J. The Atlantic spiny lumpsucker *Eumicrotremus spinosus*: life history traits and the seemingly unlikely interaction with the pelagic amphipod *Themisto libellula*. Pol Polar Res. 2013; 34: 279–287.

[53] Davenport J. Synopsis of biological data on the lumpsucker *Cyclopterus lumpus* (Linnaeus, 1758). FAO Fisheries Synopsis 147 1985; 31 pp.

[54] Berestovskiy EG. Feeding in the skates, *Raja radiate* and *Raja fyllae*, in the Barents and Norwegian Seas. J Ichthyol. 1990; 29(8): 88–96.

[55] Sorokin VP. The redfish, gametogenesis and migrations of the *Sebastes marinus* L. and *Sebastes mentella* Travin. Rapp Cons Explor Mer. 1961; 150: 245–250.

[56] Planque B, Kristinsson K, Astakhov A, Bernreuther M, Bethke E, Drevetnyak K, et al Monitoring beaked redfish (*Sebastes mentella*) in the North Atlantic, current challenges and future prospects. Aquat Living Resour. 2013; 26: 293–306.

[57] Johannesen E, Mørk HL, Korsbrekke K, Langøy H, Eriksen E, Korsbrekke K, et al. Arctic fishes in the Barents Sea 2004–2015: Changes in abundance and distribution, IMR/PINRO Joint Report Series. 2017; No. 1/2017.

[58] Sunday JM, Pecl GT, Frusher S, Hobday AJ, Hill N, Holbrook NJ, et al 2015. Species traits and climate velocity explain geographic range shifts in an ocean-warming hotspot. Ecol Lett. 2015; 18: 944e953. 10.1111/ele.12474 26189556 10.1111/ele.12474

[59] Clarke A, Johnston NM. Scaling of metabolic rate with body mass and temperature in teleost fish. J Anim Ecol. 1999; 68: 893–905.

[60] Tytell ED. Do trout swim better than eels? Challenges for estimating performance based on the wake of self-propelled bodies. Exp Fluids. 2007; 43: 701–712.

[61] Perry AL, Low PJ, Ellis JR, Reynolds JD. Climate change and distribution shifts in marine fishes. Science. 2005; 308: 1912–1915. 10.1126/science.1111322 15890845 10.1126/science.1111322

[62] Grebmeier JM, Overland JE, Moore SE, Farley EV, Carmack EC, Cooper LW, et al A major ecosystem shift in the northern Bering Sea. Science. 2006; 311: 1461–1464. 10.1126/science.1121365 16527980 10.1126/science.1121365

[63] Degen R, Vedenin A, Gusky M, Boetius A, Brey T. Patterns and trends of microbenthic abundance, biomass and production in the deep Arctic Ocean. Polar Res. 2015; 34: 24008.

[64] PINRO. The Ecosystem of the Kara Sea. PINRO Press, Murmansk 2008; 261 pp.

[65] Fetterer F, Knowles K, Meier W, Savoie M. Sea ice index. National Snow and Ice Data Center, Boulder, Colorado, USA 2002 Available online at http://nsidc.org/data/g02135.html.

[66] Dolgov AV. Annotated list of fish-like vertebrates and fish of the Kara Sea. J Ichthyol. 2013; 53: 914–922.

[67] Eriksen E, Durif CMF, Prozorkevich D. Lumpfish (*Cyclopterus lumpus*) in the Barents Sea: development of biomass and abundance indices, and spatial distribution. ICES J Mar Sci. 2014; 71: 2398–2402.

[68] Johansen GO, Johannesen E, Michalsen K, Aglen A, Fotland Å. Seasonal variation in geographic distribution of North east Arctic (NEA) cod–survey coverage in a warmer Barents Sea. Mar Biol Res. 2013; 9: 908–919.

[69] Kjesbu OS, Bogstad B, Devine JA, Gjøsæter H, Howell D, Ingvaldsen RB, et al Synergies between climate and management for Atlantic cod fisheries at high latitudes. Proc Natl Acad Sci USA. 2014; 111: 3478–3483. 10.1073/pnas.1316342111 24550465 10.1073/pnas.1316342111 PMC3948268

[70] ICES. Report of the Arctic Fisheries Working Group (AFWG), Dates 19–25 April 2016, ICES HQ, Copenhagen, Denmark. ICES CM 2016/ACOM:06. 621 pp.

