# Supplementary material for: Functional roles and redundancy of demersal Barents Sea fish: Ecological implications of environmental change
Source: PLoS One. 2018 Nov 21;13(11):e0207451. doi: 10.1371/journal.pone.0207451 (PMC6248947; doi:10.1371/journal.pone.0207451)
Supplement: S1 Table — Significant (alpha = 0.05; Bonferroni corrected alpha = 0.005) relationships in longevity among functional groups are indicated in bold. (DOCX) [file pone.0207451.s001.docx]

**S1 Table. Summary statistics of ANOVA on the species' longevity as function of functional group.** Significant (alpha = 0.05; Bonferroni corrected alpha = 0.005) relationships in longevity among functional groups are indicated in bold.

| LONGEVITY | Elasmo-branchs | Lump-  suckers | Long demersals | Semi-  pelagics | Redfish |
| --- | --- | --- | --- | --- | --- |
| Lumpsuckers | F_1,7_ = 4.042, *p* = 0.0843 |  |  |  |  |
| Long demersals | **F_1,28_ = 9.714, *p* = 0.0042** | F_1,25_ = 1.207, *p* = 0.282 |  |  |  |
| Semipelagics | F_1,12_ = 2.253, *p* = 0.159 | F_1,9_ = 1.025, *p* = 0.338 | F_1,30_ = 0.487, *p* = 0.491 |  |  |
| Redfish | **F_1,7_ = 10.6, *p* = 0.0139** | **F_1,4_ = 22.18, *p* = 0.00924** | **F_1,25_ = 80.05, *p* < 0.001** | **F_1,9_ = 25.33, *p* < 0.001** |  |
| Large demersals | F_1,20_ = 0.015, *p* = 0.904 | **F_1,17_ = 6.455, *p* = 0.0211** | **F_1,38_ = 19.35, *p* < 0.001** | **F_1,22_ = 4.584, *p* = 0.0436** | **F_1,17_ = 18.17, *p* < 0.001** |
