# Supplementary material for: Functional roles and redundancy of demersal Barents Sea fish: Ecological implications of environmental change
Source: PLoS One. 2018 Nov 21;13(11):e0207451. doi: 10.1371/journal.pone.0207451 (PMC6248947; doi:10.1371/journal.pone.0207451)
Supplement: S2 Table — Significant (alpha = 0.05; Bonferroni corrected alpha = 0.005) relationships in longevity among functional groups are indicated in bold. (DOCX) [file pone.0207451.s002.docx]

**S2 Table.** **Summary statistics of ANOVA on the species' trophic level (TL) as function of functional group.** Significant (alpha = 0.05; Bonferroni corrected alpha = 0.005) relationships in longevity among functional groups are indicated in bold.

| TROPHIC LEVEL | Elasmo-branchs | Lump-suckers | Long demersals | Semi-pelagics | Redfish |
| --- | --- | --- | --- | --- | --- |
| Lump-suckers | F_1,7_ = 1.711, *p* = 0.232 |  |  |  |  |
| Long demersals | **F_1,28_ = 10.88, *p* = 0.00265** | F_1,25_ = 0.151, *p* = 0.701 |  |  |  |
| Semi-pelagics | F_1,12_ = 1.219, *p* = 0.291 | F_1,9_ = 0.027, *p* = 0.873 | F_1,30_ = 0.678, *p* = 0.417 |  |  |
| Redfish | F_1,7_ = 0.152, *p* = 0.708 | F_1,4_ = 6.759, *p* = 0.0601 | **F_1,25_ = 13.87, *p* = 0.001** | F_1,9_ = 1.422, *p* = 0.263 |  |
| Large demersals | F_1,20_ = 0.213, *p* = 0.649 | F_1,17_ = 0.849, *p* = 0.37 | **F_1,38_ = 8.141, *p* = 0.00697** | F_1,22_ = 0.884, *p* = 0.357 | F_1,17_ = 0.522, *p* = 0.48 |
