# Supplementary material for: Functional roles and redundancy of demersal Barents Sea fish: Ecological implications of environmental change
Source: PLoS One. 2018 Nov 21;13(11):e0207451. doi: 10.1371/journal.pone.0207451 (PMC6248947; doi:10.1371/journal.pone.0207451)
Supplement: S3 Table — Significant (alpha = 0.05; Bonferroni corrected alpha = 0.005) relationships in longevity among functional groups are indicated in bold. (DOCX) [file pone.0207451.s003.docx]

**S3 Table. Summary statistics of ANOVA on the species' maximum body length (ML) as function of functional group.** Significant (alpha = 0.05; Bonferroni corrected alpha = 0.005) relationships in longevity among functional groups are indicated in bold.

| MAXIMUM BODY LENGTH | Elasmo-branchs | Lump-suckers | Long demersals | Semi-pelagics | Redfish |
| --- | --- | --- | --- | --- | --- |
| Lumpsuckers | **F_1,7_ = 13.05, *p* = 0.00859** |  |  |  |  |
| Long demersals | **F_1,28_ = 11.59, *p* = 0.00202** | F_1,25_ = 0.349, *p* = 0.56 |  |  |  |
| Semipelagics | **F_1,12_ = 6.285, *p* = 0.0276** | F_1,9_ = 1.686, *p* = 0.226 | F_1,30_ = 0.85, *p* = 0.364 |  |  |
| Redfish | F_1,7_ = 4.631, *p* = 0.0684 | F_1,4_ = 2.074, *p* = 0.223 | F_1,25_ = 0.373, *p* = 0.547 | F_1,9_ = 0, *p* = 0.999 |  |
| Large demersals | F_1,20_ = 0.015, *p* = 0.904 | F_1,17_ = 2.433, *p* = 0.137 | **F_1,38_ = 10.46, *p* = 0.00252** | F_1,22_ = 2.497, *p* = 0.128 | F_1,17_ = 0.976, *p* = 0.337 |
